# The emerging applications and advancements of Raman spectroscopy in pediatric cancers

**DOI:** 10.3389/fonc.2023.1044177

**Published:** 2023-02-06

**Authors:** Chenbei Li, Chengyao Feng, Ruiling Xu, Buchan Jiang, Lan Li, Yu He, Chao Tu, Zhihong Li

**Affiliations:** ^1^ Hunan Key Laboratory of Tumor Models and Individualized Medicine, The Second Xiangya Hospital, Central South University, Changsha, China; ^2^ Department of Orthopaedics, The Second Xiangya Hospital, Central South University, Changsha, Hunan, China; ^3^ Department of Pathology, The Second Xiangya Hospital, Central South University, Changsha, Hunan, China; ^4^ Department of Radiology, Second Xiangya Hospital of Central South University, Changsha, Hunan, China

**Keywords:** Raman spectroscopy, pediatric cancer, application, diagnosis, treatment

## Abstract

Although the survival rate of pediatric cancer has significantly improved, it is still an important cause of death among children. New technologies have been developed to improve the diagnosis, treatment, and prognosis of pediatric cancers. Raman spectroscopy (RS) is a non-destructive analytical technique that uses different frequencies of scattering light to characterize biological specimens. It can provide information on biological components, activities, and molecular structures. This review summarizes studies on the potential of RS in pediatric cancers. Currently, studies on the application of RS in pediatric cancers mainly focus on early diagnosis, prognosis prediction, and treatment improvement. The results of these studies showed high accuracy and specificity. In addition, the combination of RS and deep learning is discussed as a future application of RS in pediatric cancer. Studies applying RS in pediatric cancer illustrated good prospects. This review collected and analyzed the potential clinical applications of RS in pediatric cancers.

## 1 Introduction

Pediatric cancer is uncommon but represents a significant cause of disease-related death among children (1–3). Pediatric cancers represented only 2% of all reported cancer cases but accounted for 10% of child deaths (4, 5). Leukemia (30–40%), central nervous system (CNS) tumors (20%), lymphoma, and osteosarcoma (5%) are the most common pediatric cancers in children aged 0–19 years (6, 7). Two peak incidences of pediatric cancers occur before the age of two and during adolescence (8). Leukemia frequently occurs throughout childhood, while CNS tumors account for most diagnoses in children under two years. Meanwhile, the incidence of OS and lymphoma increases steeply after nine years (5, 9). In contrast to adult cancers, pediatric cancers have more significant influence on children in the long term (4). Pediatric cancer patients may die from cancers or their complications (10). Technologies focusing on pediatric cancer have significantly improved in the last few decades, and survival has increased by over 60% (11, 12). However, compared to adult cancer, there are approved treatments for pediatric cancer. Once the patients grow up, side effects and complications become more severe (13). Thus, new perspectives on pediatric cancer are urgently needed to address these challenges doctors face.

Raman spectroscopy (RS) is an optical spectroscopic technique using different frequencies of scattering light to characterize biological specimens (14, 15). It probes the vibrational modes related to chemical bonds in a sample and obtains the “Raman Spectrum”, which is a unique spectral fingerprint of the sample (16, 17). Different samples possess their chemical compositions, which give RS the ability to reveal changes in the components and structure of target samples in a non-destructive manner (18). It provides deep insights into biological activities, offering a new angle to analyze diseases and assisting in diagnosis, treatment, and prognostic evaluation (15, 19). In fact, vibrational spectroscopy has been quite active over the past two decades. It is considered to be used for both *in vivo* and ex vivo disease diagnosis (20). Moreover, it is applied in multitudinous areas, such as characterization of tumor margins in surgery (21, 22), disease detection using *in vivo*-endoscopic probe (23), and drug screening (24, 25). Therefore, RS is highly valued for clinical application in pediatric cancers and provides critical biological information for doctors (26). This review summarizes articles on RS and pediatric cancers within the last two decades and explores the clinical role of RS in pediatric cancers.

## 2 Raman spectroscopy

### 21 Principle of Raman spectroscopy

**Figure 1:**
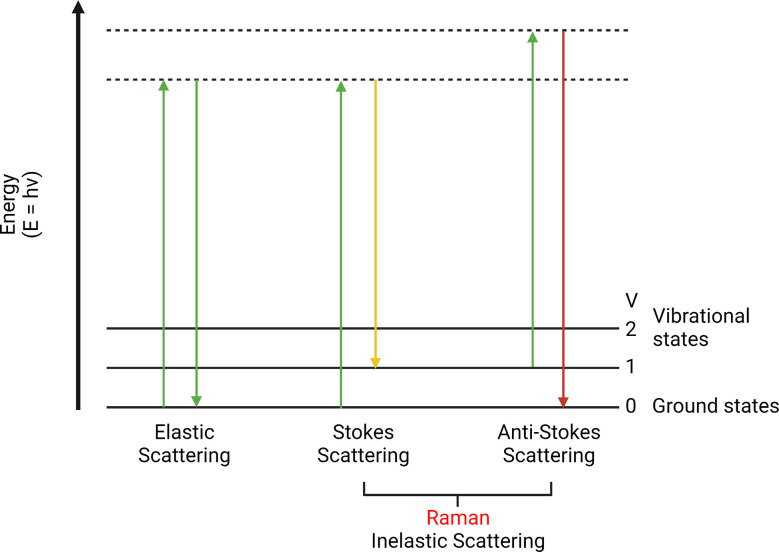
Elastic scattering, Stokes, and anti-Stokes Raman scattering. This is a scattering energy level diagram (molecular energy states). Stokes scattering leads to an energy shift from incident photons to chemical bonds. Anti-Stokes scattering moves vibrational excited chemical bonds to the incident light.

The Raman effect was first discovered by the physicist C.V. Raman and his team in 1928 (27, 28). They observed the absorption and scattering of photons passing through a medium. If a photon is absorbed by a molecule, the molecule will gain its energy. However, if a photon is scattered and its energy remains the same, it is called “elastic scattering”; otherwise, it is called “inelastic scattering”. In addition, the inelastically scattered photons with higher energy are called anti-Stokes Raman scattering, while those with lower energy are Stokes Raman scattering (29) (**Figure 1**).

This little energy difference is known as the Raman effect (or shift). It is a rare phenomenon that only happens in approximately one in 10^7^ of the incident photons (30, 31). This energy difference can be used to detect different chemical bonds to identify molecular structures (32, 33). Since the photon energy is inversely proportional to the wavelength, Raman shift can perform a color shift named the Raman spectrum. It is a plot of the intensity of the scattered light under different wavenumber (cm^-1^) (34). This plot is associated with a particular vibrational mode of the molecule and is directly proportional to its concentration. It is a summary of all Raman active molecules in a sample, such as proteins, lipids, etc. The range below 2000 cm^-1^ is usually called the fingerprint region, which is composed of unique Raman signals biological molecules (35, 36). By analyzing the Raman spectrum, it is possible to detect specific molecules and distinguish pathological tissues (37, 38). Moreover, biological activities inside normal or diseased tissues can also be revealed (39, 40).

### 2.2 Advantages and disadvantages of Raman spectroscopy

**Figure 2:**
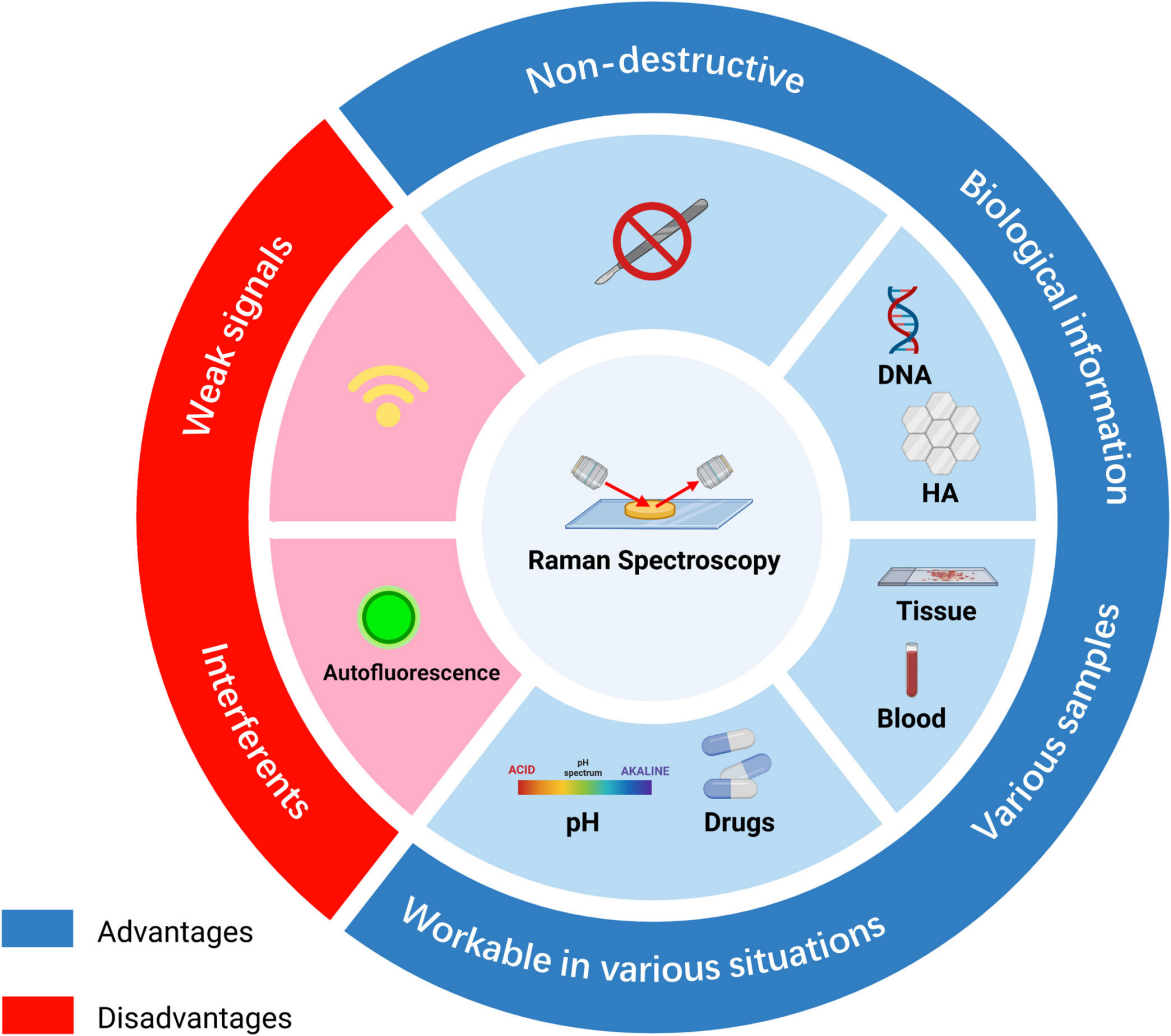
Summary of the advantages and disadvantages of Raman spectroscopy in diagnosis. RS possesses non-destructive properties and is workable under various environments, such as different pH or drug conditions. RS can be used to analyze tissue, blood, or supernatant samples and provides biomolecular information on DNA or biomarkers. However, signals of RS are weak and can be affected by other interferents, such as fluorescence. HA, hydroxyapatite.

RS has advantages in clinical application, although its function is restricted by some limitations. The key pros and cons of RS are listed below (**Figure 2**). The most common advantage is its non-destructive and label-free properties (41, 42). Since the influence of water in RS is less than in other spectroscopies, various samples can be investigated, including biological fluids, cell cultures, tissue sections, and organs (43). After the acquisition of samples, cellular biological activities and chemical components can be detected, which are suitable for chemical analysis or cell classification (44). In addition, RS can be applied to *in vivo* detection to monitor disease stage or the response of cells under different situations, including drugs and pH (45).

Nevertheless, there are some shortcomings. Due to the low probability of occurrence of Raman scattering, the signals of RS are weak, limiting the sensitivity of RS (46). In addition, weak Raman signals lead to a long acquisition time, and the interference of autofluorescence may make the signal difficult to discern (47) (Schie et al.) RS results mostly depend on the identification of spectral peaks. The accuracy of the identification has a significant influence on the final results. Most researchers identified RS data based on previously published articles. Thus, without a database containing most of the required peaks in the spectral range, peak identification can be time-consuming (36). Unlike clinical biochemical examinations, RS requires both normal and abnormal tissue for comparison, and data on RS often require additional analysis (48).

### 2.3 Differences between Raman spectroscopy and other spectroscopies

Infrared spectroscopy (IRS) and fluorescence spectroscopy (FS) are powerful techniques in the clinical medical field. They have special characteristics compared to RS (**Table 1**).

**Table 1:** Characteristics of different spectroscopy.

	Raman Spectoscopy	IR	Fluorescence
**Region (wavelength [µm])**	2.5-200	0.75-1000	0.18-0.8
**Mechanism**	Inelastic photon scattering	Absorption of specific frequencies of light	Absorption and emission
**Problems and challenges**	1. Raman signal affected by autofluorescence. 2. Long acquisition time.	1. Signal affected by water absorbance. 2. Influencing signal from H2O and CO2.	1. Not suitable for intransparent samples. 2. Only works on molecules that absorb excitation light.

IR, infrared.

IRS and RS belong to a molecular vibration area. RS is based on photon scattering, while IRS measures regions where the discrete energy of molecules is related to vibration. The absorption of photons in the electromagnetic spectrum was monitored and can be used to discriminate different bonds in samples (49–51). In the IR region, water absorbance is so strong that it could affect the collection and analysis of IR spectra. However, Raman regions possess weak water signals, which are more accurate for *in vivo* analysis.

FS has been applied in various diseases, such as breast cancer (52) and cervical cancer (53, 54). It measures the concentration of a substance in a solution based on fluorescent properties, which is directly proportional to the intensity of emitted light (55, 56).

### 2.4 Raman-based technologies

**Figure 3:**
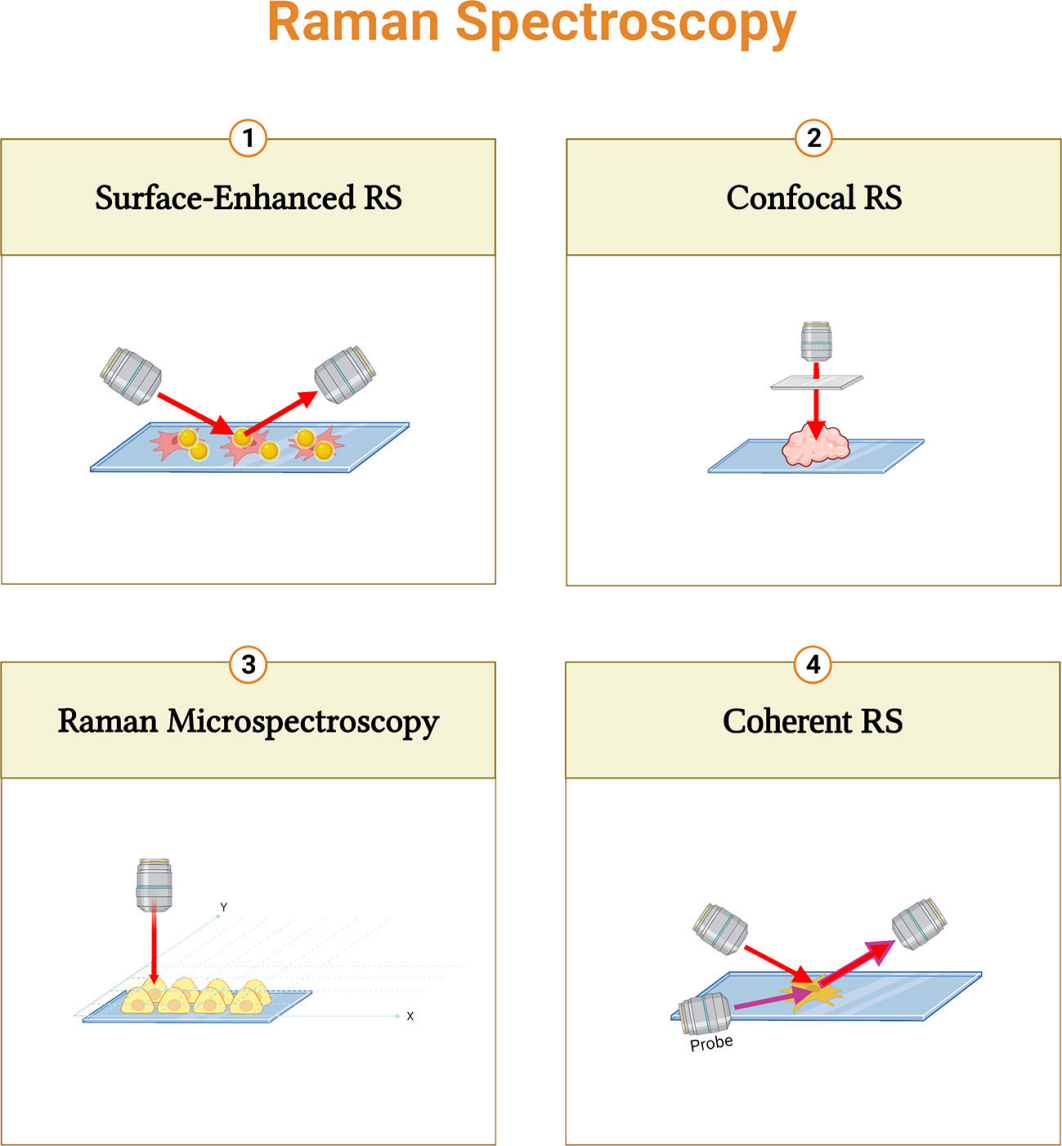
Summary of different Raman spectroscopy techniques. Surface-enhanced RS (SERS) performs enhanced signals with metal nanoparticles; Confocal RS is a combination of confocal microscope and RS; Raman micro-spectroscopy combines RS with microscope; and Coherent RS is RS with two light sources.

Various types of enhanced Raman techniques were developed to intensify the sensitivity of detection and speed up imaging production (57, 58) (**Figure 3**).

Raman micro-spectroscopy is a non-destructive technique that combines RS with an optical microscope. It provides correlations between biochemical and morphological properties (59, 60). Furthermore, the combination of a confocal microscope and RS can improve the depth of information. (61, 62). In Coherent RS, vibrational modes are generated by two light sources. It focuses on specific molecular bands that shorten the acquisition time (63). Moreover, surface-enhanced RS (SERS) performs enhanced signals with metal nanoparticles to increase the sensitivity (64, 65).

### 2.5 Data analysis of Raman spectra

Suitable multivariate analysis is crucial to make a diagnosis more accurate. Data of Raman spectra are composed of multiple bands and peaks that represent different molecules. In addition, the dataset of Raman spectra contains intensity and wavenumber, and it is correlated with diseases and cell types (66). Therefore, multivariate analysis is necessary to simplify the data using statistical algorithms such as principle component analysis (PCA). PCA reduces the dimension of data, and most of the variation is unchanged (67, 68). This reduction is completed by identifying the principal components. It can simplify the data by presenting a few samples instead of a large number of redundant data. (69). Combining PCA with other statistical methods can be used for discriminating between diseases, cell states, and cell types (70). In general, multivariate analysis can be divided into two classes: supervised and unsupervised, based on targets and samples (71). Meanwhile, hierarchical clustering analysis (HCA) (72), k-means clustering (KMC) (73, 74), and linear discriminant analysis (LDA) (75, 76) are supervised methods. Their purpose is to explore, and no previous information about the samples is needed. On the other hand, unsupervised methods focus on pattern recognition based on existing labels like disease diagnosis or cell type classification. Unsupervised methods include algorithms like PCA, multiple linear regression (77, 78), and partial least squares (PLS) (79).

## 3 The roles of Raman spectroscopy in pediatric cancer

This review is a collection of articles from the PubMed database from 2012 to 2022 (**Table 2**). The search terms were Raman spectroscopy, osteosarcoma, acute lymphoblastic leukemia, acute myeloid leukemia, lymphoma, and glioma.

**Table 2:** Summary of RS studies on pediatric cancers.

RS Techniques	Sample	Diagnostic performance	Reference	Advantages of RS	Disadvantages of RS
**Osteosarcoma**				1. Early and non-destructive diagnosis 2. Can be universally applied in patients. 3. Samples are easy to be acquired. 4. Tumor margin evaluation.	1. Lack of specific marker for RS diagnosis. 2. Lack of RS OS database. 3. Time consuming in analyzing tissue samples. 4. RS data analysis is complex.
SERS	*In vitro*, 143B, HOS, MG63 cells; Plasma of patients (n=20) and control (n=20)	PLS-DA model accuracy: 95%, sensitivity: 100%, Specificity: 90%.	(80)		
RS	*In vitro*, five different cell lines (MSC, hFOB, MG63, SaOS2, and 143B)	Not mentioned.	(81)		
Confocal RS	*In vitro*, Undifferentiated MSCs, Osteo-differentiated MSCs, MG-63 cells	Not mentioned.	(82)		
Confocal RS	*In vitro*, K7M2 cells	PCA-SVM model accuracy: 91.7%(24h), 90%(48h).	(83, 84)		
Confocal RS	*In vitro*, osteosarcoma compact bone (n=10), trabecular bone (n=5), control compact bone (n=1)	Not mentioned	(85)		
**ALL and AML**				1. Rapid and economic diagnosis method. 2. Reveal biomolecule information.	1. Lack of RS ALL/AML database. 2. RS data analysis is complex.
Confocal RS	*In vitro*, fresh blood sample of ALL (n=5), AML (n=4), CML (n=3) and control (n=21)	PCA-LDA model in leukemia detection and classification sensitivity: 100%, specificity:100%	(86)		
RS	*In vitro*, whole blood sample of leukemia (n=17) and control (n=21). Plasma of leukemia (n=15) and control (n=25).	PLS model of whole blood sensitivity: 91.9%, specificity: 100%, accuracy: 96.5%. PLS model of plasma sensitivity: 95.7%, specificity: 98%, accuracy: 97.1%.	(87)		
RS	*In vitro*, bone marrow supernatant of AML (n=61), ALL(n=22) and control (n=5).	OPLS-DA model sensitivity: 85%, specificity: 90%.	(88)		
RS	*In vitro*, MN60 cells from ALL patients, leukocyte from control (n=3)	PCA model accuracy: 99%, PCA-LDA model accuracy: 98.6%.	(89)		
SERS	*In vitro*, bone marrow samples from AML-M0 (n=13), AML-M2 (n=20), AML-M3 (n=31), AML-M4 (n=2), AML-M5 (n=89).	Not mentioned	(90)		
SERS	*In vitro*, cell line (THP-1, HaCaT), DNA sample from AML (n=17), control (n=17).	PCA-LDA model accuracy: 82.2%, SVM model accuracy: 75.3%	(91, 92)		
Confocal RS	*In vitro*, AML-M0 (n=2), AML-M2 (n=2), AML-M3 (n=2), AML-M6 (n=1).	PCA-LDA model accuracy: 98%.	(93)		
Raman micro spectroscopy	*In vitro*, cell line (HL60, K562)	Classification accuracy of BaP treated cells: 95.23%, accuracy of control cells: 89.11%.	(94)		
SERS	*In vitro*, bone marrow supernatant of AML	Prognosis prediction model accuracy: 84.78%.	(95)		
**Lymphoma**				1. Simple and non-destructive diagnosis. 2. Samples are easy to be acquired.	1. Equipment of RS are expensive. 2. RS data analysis is complex. 3. Equipment is costly. Hard to promote in some area.
SERS	*In vitro*, blood sample of DLBCL stage I (n=19), stage II (n=19), stage III (n=9), stage IV (n=6) and control (n=47).	Classification model accuracy: 87.3%, sensitivity: 92.1%, specificity: 80.9%. Discrimination of early and late-stage model accuracy: 90.6%.	(96)		
SERS	*In vitro*, lymph node tissue sample of BCL (n=9), TCL (n=9), Met (n=10) and control (n=10).	PCA-QDA discrimination model accuracy: 94.7%.	(97)		
RS	*In vitro*, DLBCL cell line (KML1, A4/Fuk, HF), leukocyte from control.	Discrimination sensitivity: 74-94%, specificity: 90-100%.	(98)		
RS	*In vitro*, blood plasma of DLBCL (n=33), CLL (n=39), control (n=30).	OPLS-DA CLL model sensitivity: 92.86%, specificity: 100%. OPLS-DA DLBCL model sensitivity: 80%, specificity: 92.31%.	(99)		
**Glioma**				1. Guide the resection of tumor. 2. No preparation and special staining are needed. 3. Real-time diagnosis.	1. RS has limited field of view. 2. RS data analysis is complex.
RS	*In vitro*, samples from glioma patients (n=4), healthy brain astrocytes.	Discrimination model accuracy: 92.5%.	(100)		
Confocal RS	*In vitro*, blood sample from glioma patients (n=38), control (n=45).	PLS-LDA model accuracy: 97.87%, sensitivity: 98.1%, specificity: 98.19%.	(101)		
SERS	*In vitro*, tissue samples from astrocytoma (n=2), GBM (n=3), control (n=5).	Discrimination accuracy: 96%	(102, 103)		
RS	*In vivo* detection of brain tumor cells.	Discrimination sensitivity: 93%, specificity: 91%.	(104)		
RS	*In vitro*, tissue sample from pediatric medulloblastoma (n=4), glioma (n=19) and control (n=5).	Glioma identification accuracy: 96.7%, medulloblastoma identification accuracy: 93.9%. Tissue level classification accuracy: 100%.	(105)		
RS	*In vitro*, samples from GBM, medulloblastoma, meningioma and control.	Discrimination model sensitivity: 97.4%, specificity: 100%.	(106)		
Raman micro-spectroscopy	Human GBM cell line (U-251)	Not mentioned	(107)		

RS, Raman spectroscopy; SERS, surfaced enhanced Raman spectroscopy; PCA, principal component analysis; HCA, hierarchical cluster analysis; LDA, linear discriminant analysis; PLS, partial least squares; OS, osteosarcoma; hFoB, human fetal osteoblast; hMSC, human bone marrow-derived mesenchymal stem cells; HA, hydroxyapatite; MSC, mesenchymal stomal cells; ALL, acute lymphoblastic leukemia; AML, acute myeloid leukemia; CML, chronic myeloid leukemia; NHL, Non-Hodgkin lymphoma; HL, Hodgkin lymphoma; NK, natural killer; DLBCL, diffuse large B-cell lymphoma; Met, melanoma; OPLS-DA, orthogonal partial least squares discriminant analysis; SVM, support vector machine; BCL, B-cell lymphoma; TCL, T-cell lymphoma; Met, melanoma; CLL, chronic lymphocytic leukemia; GBM, glioblastoma.

### 3.1 Osteosarcoma

**Figure 4:**
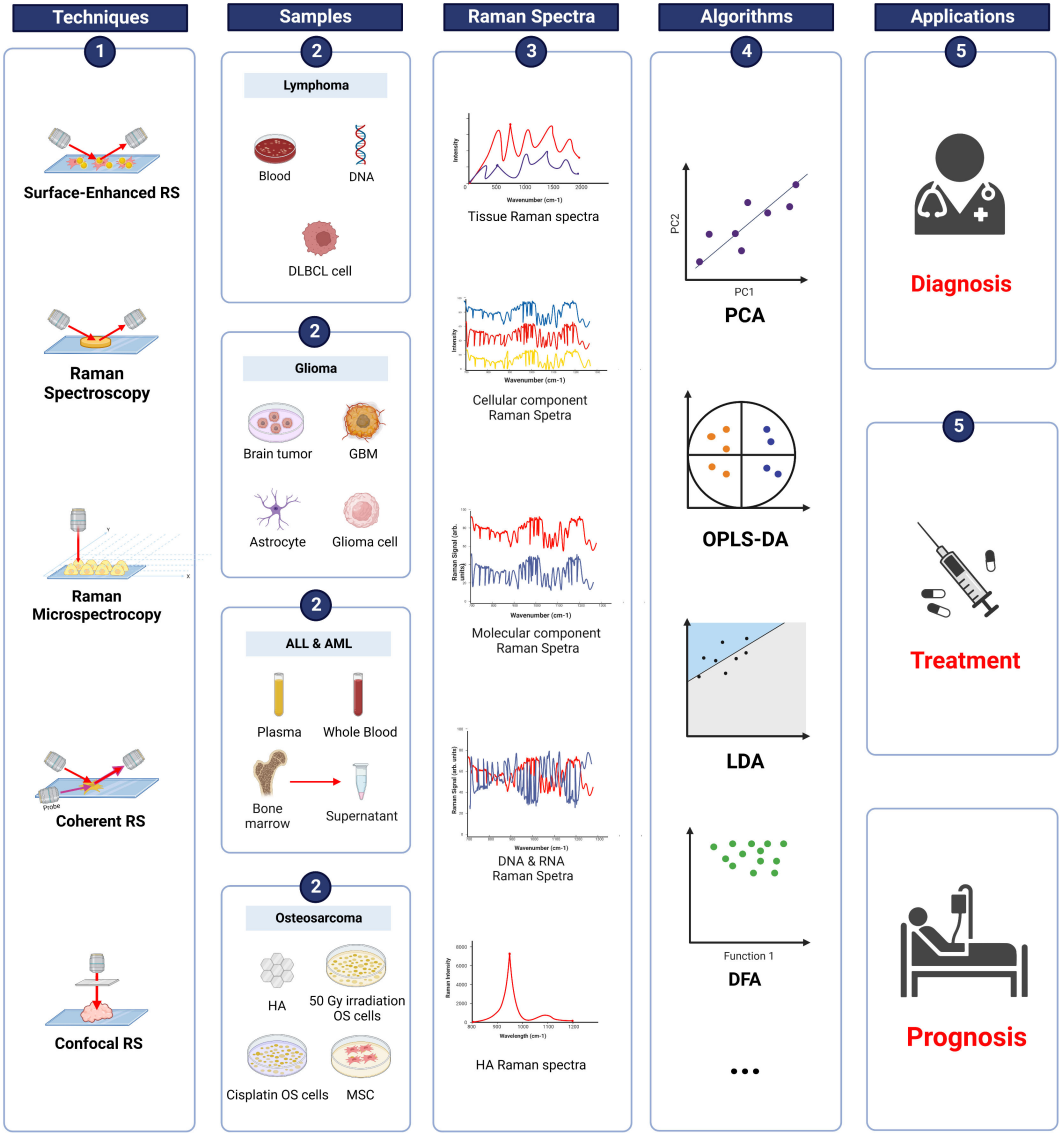
Raman spectroscopy techniques in pediatric cancers. Summary of information on RS in osteosarcoma, ALL, AML, lymphoma, and glioma. RS techniques were applied to various samples (blood, tissue, supernatant etc.) and produced Raman spectra. Data were analyzed using different algorithms, such as PCA, OPLS-DA, and LDA. The results demonstrated the potential of applying RS in the diagnosis, treatment, and prognosis of pediatric cancers. DLBCL, diffuse large B-cell lymphoma; GBM, glioblastoma; ALL, acute lymphoblastic leukemia; AML, acute myeloid leukemia; HA, hydroxyapatite; OS, osteosarcoma; MSC, mesenchymal stem cells; PCA, principal component analysis; OPLS-DA, orthogonal partial least squares discriminant analysis; LDA, linear discriminant analysis; DFA, discrimination function analysis.

Osteosarcoma is the most common malignant bone tumor in children (108, 109), accounting for 20% of all bone malignancies (110, 111) and 2.5% of pediatric malignancies (112). According to Surveillance, Epidemiology, and End Results (SEER) data from 1973 to 2004, the incidence of osteosarcoma has two peaks. The first peak occurs between 10 and 14 years of age, while the second peak is observed after 60 (113, 114). OS in adolescence commonly develops in the metaphysis of long bones, such as the distal femur and proximal tibia (115–117). With the improvement of technology, the survival rate of OS has significantly increased from less than 20% to around 70% (118). The number of studies exploring the clinical application of Raman spectroscopy in OS within the last few decades continues to increase. The applications of RS in OS are summarized in **Figure 4**.

Current pediatric osteosarcoma diagnosis, prognosis and treatment decisions are composed of clinical manifestation, CT, MRI scan, biopsy, and laboratory results (119). CT and MRI are considered the most common and convenient diagnostic techniques (120). A study indicated that the accuracy of CT and MRI in detecting bone lesions of pediatric osteosarcoma was 67% and 86% (121). Accurate clinical diagnosis of osteosarcoma requires biopsies. However, it takes time and requires complex operations to get precise the tumor tissue. To improve the efficiency of diagnosis, Han’s team isolated exosomes from plasma and profiled multiple biomarkers for the diagnosis of osteosarcoma (80). An exosome is a small vesicle that carries nucleic acids, proteins, and lipids (122, 123). It transmits information and plays an important role in tumorigenesis (124, 125). In Han’s study, Raman bands at 1000, 1075, and 1375 cm^−1^ (CD63, vimentin (VIM), and epithelial cell adhesion molecule (EpCAM)) in exosomes of osteosarcoma patients were significantly higher. The area under the receiver operating characteristic curve was 0.971, and the sensitivity, specificity, and accuracy of the model in identifying osteosarcoma was 100%, 90%, and 95% (80).

Osteosarcoma results from a disorder in the differentiation procedures of mesenchymal stem cells (MSC) (126). Minerals like hydroxyapatite (HA), a mineral form of calcium phosphate, are highly expressed in osteoblast differentiation of MSCs and can be measured using RS (127). Malignant cancer cells are immature and produce less HA (128). Thus, it is possible to use HA as a potential biomarker for grading differentiation. Chiang et al. developed an RS-based measurement to grade *in vitro* osteosarcoma cells based on HA level. A significant difference in the peak intensity of symmetric stretching of phosphate groups (around 960 cm^-1^), which represents the expression of HA in cells, was observed in Raman spectra of living human fetal osteoblast (hFoB), human bone marrow-derived mesenchymal stem cells (hMSC), low-grade (MG-63), and high-grade (SaOS2, 143B) osteosarcoma cells. Results showed that HA was highly expressed in MG63 cells but not in hMSC and SaOS2 and 143B cells (81). Based on a previous study, D’Acunto et al. also employed RS to detect HA in MG-63 cells and MSCs. The identification was based on HA Raman peaks (around 960 cm^-1^) with the support of multivariate analysis, and the results suggested that HA Raman peaks are greater in MG-63 cells (82). Moreover, the expression of matrix metalloproteinases (MMP2, MMP9) was rich in high-grade but negligible in low-grade osteosarcoma. MMP is an angiogenesis factor closely related to the invasion and metastasis of tumors (129). The upregulation of MMP is correlated with poor prognosis in osteosarcoma patients (130). Generally, MMP was measured using RT-PCR, and the expression level was positively correlated with HA levels. This may highlight the inverse correlation between the HA level and the prognosis of osteosarcoma. Current evaluation of the maturation of osteogenic differentiation is based on histochemical and molecular biological methods, such as Alizarin red staining, western blot, and RT-PCR (131, 132). However, these assays are time-consuming and cannot be applied on live cells. The detection of HA production using RS could be a rapid method for grading OS. During surgery, accurate resection of tumor is extremely crucial for patients’ prognosis. Yet, precise information of margin offered by biopsy is time-consuming. In addition, CT and MRI are unable to distinguish osteosarcoma cells from normal cells. In this case, RS is suitable for rapid detection of osteosarcoma cells in the resection margins.

A combination of chemotherapy and surgery is the conventional therapeutic approach for pediatric osteosarcoma (133). Cisplatin is a drug for osteosarcoma chemotherapy and the first platinum-based anti-tumor drug widely used to treat cancers (134). Drug resistance by osteosarcoma is a problem that affects patients’ prognosis (135). In this regard, a better understanding of cellular drug interactions is needed for therapeutic improvement. Wang et al. selected RS as an analytical technique for real-time extraction of biochemical information from living cells, and spectra of *in vitro* K7M2 osteosarcoma cells treated with 20 μm and 40 μm were measured. The results suggested that the major biochemical changes induced by cisplatin in osteosarcoma cells are on protein and nucleic acid. Raman bands of nucleic acids (766, 1096, 1248, and 1323 cm^−1^) were decreased after treatment with cisplatin. Meanwhile, Raman bands of cisplatin-induced apoptosis (657, 1002, 1248, 1450, and 1660 cm^−1^) showed the most significant spectral features. The results indicate that cisplatin mainly targets the nucleus and affects the secondary structure of proteins (83, 84).

Extra Corporeal irradiation and Reimplantation Therapy (ECRT) is an established biological reconstruction technique of limb salvage surgery that mainly focuses on the treatment of malignant bone tumors (osteosarcoma, Ewing sarcoma etc.) and presents good clinical outcomes (136, 137). Specifically, the resected bone of patients will be reimplanted after high-dose irradiation (50 Gy to 300 Gy). To enrich the understanding of compositional and structural changes of bone under high-dose irradiation, Chauhan and team investigated Raman spectra of human bone of fifteen osteosarcoma and Ewing sarcoma patients on osteosarcoma treatment with 50 Gy single-dose irradiation. Thirteen patients were under 18 years, and three were between 18 and 26 years. Pre- and post-irradiation samples were scanned by confocal Raman spectroscope using a 785 nm excitation laser at 50% laser power. Thirty accumulations were taken at the scan area, and the average was analyzed. The evaluation of bone quality was conducted using calcium content and mineral to matrix ratio, and change in collagen was quantified with the deconvolution of Amide I peak. The findings showed that a 50 Gy dose of radiation resulted in the loss of approximately 50% of mineral components. Thus, irradiation might cause changes in bone composition, which have negative effects on bone quality (85). This provides valuable insights for clinicians in predicting the outcome of high-dose irradiation and designing irradiation doses for treatment.

Early diagnosis of osteosarcoma mainly depends on imageology results. RS possesses advantages in screening patients using their biochemical information, and it is able to help doctors to determine resection margins, which can largely reduce recurrence. In addition, RS is universally applicable in pediatric patients since samples for RS analysis are easy to acquire. Tumor biopsy is a destructive and painful process for patients, and RS is an ideal replacement for biopsy. However, RS has its limitations. Current RS can only be the reference for clinicians as the accuracy of RS needs to be improved. Mineralization level is a biomarker of bone-related diseases. Thus, the future direction of RS can focus on detecting mineralization levels and discovering specific markers for osteosarcoma.

### 3.2 Acute lymphoblastic leukemia

Acute lymphoblastic leukemia (ALL) is a malignancy derived from B/T cell lineage lymphatic progenitor cells during the transformation of B/T lymphocytes (138, 139). It is the most common malignancy of childhood and mainly develops in children aged 2–5 years (140). The 5-year survival rate of children with ALL is as high as 80–90% (141, 142). However, the prognosis of ALL recurrence is still very poor (143). Thus, a more accurate classification of ALL is needed to reduce recurrence and improve the clinical outcome of ALL. In recent years, studies have aimed at discovering the application of RS in ALL in multiple areas. The applications of RS in ALL are briefly summarized in **Figure 4**.

The diagnosis of acute leukemia, including ALL and acute myeloid leukemia (AML), mainly depends on clinical symptoms and peripheral blood or bone marrow aspiration examinations. Further classification is based on cell immunology, flow cytometry, and molecular biology. However, these diagnostic methods are time-consuming and costly (87). Therefore, low-cost and rapid diagnostic methods are needed for early definition and classification of leukemia (144, 145).

Confocal Raman microscope with a laser of 830 nm and power of 17 mW was applied on fresh blood serum from different types of leukemia patients (Ages 8–50 years) and healthy volunteers to detect and identify leukemia. The msain differences between leukemia and control spectra were at 1,338 (tryptophan (Trp), α-helix, and phospholipids), 1,447 (lipids), 1,523 (β-carotene), 1,556 (Trp)), 1,587 (protein, tyrosine (Tyr)), 1,603 (Tyr, phenylalanine (Phe)), and 1,654 (proteins, amide I, α-helix, phospholipids) cm^−1^. Spectral information was analyzed using PCA and LDA for discrimination and identification of leukemia, and the prediction outcome using RS was validated using pathological diagnosis. The results showed the sensitivity and specificity of leukemia detection and discrimination were 100%. (86). Current diagnosis of leukemia is mainly based on cytomorphology and immunophenotyping (146, 147). The accuracy of phenotyping diagnosis is 94% (146), and the specificity and sensitivity of flow cytometry are 98% and 95.7% (147). Diagnosis based on RS is close or even better. RS was applied to whole blood samples to identify spectral differences based on chemical components like proteins, amino acids, and lipids. PLS discrimination model based on whole blood spectra showed a sensitivity of 91.9%, specificity of 100%, and accuracy of 96.5% in discriminating leukemia from control. The classification of leukemia subtypes using plasma had a sensitivity, specificity, and accuracy of 95.7%, 98%, and 97.1% (87).

According to a previous study, Raman peaks of Trp, Tyr, Phe, phospholipid, and β-carotene contribute to the diagnosis of leukemia. For further diagnosis, Liang’s team established a detection method for AML and ALL, using bone marrow supernatant from 22 patients with ALL (aged 3–55 years), 61 patients with AML (aged 1–69 years) and five healthy donors (aged 1–40 years). Raman peaks of 1437, 1443, and 1579 cm^-1^ (cholesterol, high-density lipoprotein (HDL), low-density lipoprotein (LDL), adenosine deaminase (ADA), and hemoglobin) were used for diagnosis. The results indicated that the sensitivity of RS analysis was 85% and 90% specificity based on bone marrow supernatant (148). Another article published in 2018 used RS to identify leukocyte subpopulations of lymphocyte (B, T, and natural killer (NK) cells), monocyte, and granulocytes. Spectral differences between monocytes and lymphocytes were nucleic acid and protein bands. In addition, spectral differences around 833, 977, 1337, 1547, and 1617 cm^−1^ were found in granulocyte. Classification based on Raman data provides 99% accuracy. Moreover, they proved that RS can effectively monitor the cell response to low-dose chemotherapy. Methotrexate (MTX)-treated (0.01, 0.1, and 1 μm, 72 h) cells presented a decreasing trend of RS intensities. In addition, proteins and nucleic acids decreased as the concentration of MTX increased (89).

### 3.3 Acute myeloid leukemia

AML is a group of aggressive hematopoietic malignancies that arise from myeloid precursors (149). Although AML treatments have improved significantly in recent decades, relapses of AML and drug resistance are still the major challenges in the treatment of AML (150), with only 40% of young patients staying in remission at five years after treatment (151, 152). Therefore, more accurate classification and diagnosis are needed. Advanced technology such as RS can be applied in AML to improve outcome. A description of RS techniques in AML is summarized in **Figure 4**.

The most recent study of AML and RS was conducted in 2022. SERS with Ag nanoparticles was used to detect biochemical varieties of blood serum samples from patients aged 16–60 years. Significant differences were found in amino acids and proteins, which can be used as SERS biomarkers to differentiate AML subtypes. Raman bands at 495, 725, 1002, 1070, 1616, and 1653 cm^−1^ (amino acid, nucleic acid, and protein) demonstrated significant differences in samples. Quadruple detection of a combination of Raman band ratios 533/1002, 1070/1653, 725/1653, and 1616/1653 could be used as a biomarker for the primary diagnosis of AML (90). Besides, some articles mentioned above in the ALL section (86, 87, 148) also demonstrated the potential of RS in the diagnosis and classification AML.

Various diagnostic models have been established based on RS techniques to obtain a rapid and accurate classification of AML. Low methylation of DNA can be used to detect cancer (153). DNA from *in vitro* AML cells were extracted and compared to normal DNA. Raman spectra illustrated the 1005 cm^-1^ band of 5-methylcytosine decreased, and classification based on this band demonstrated 82% accuracy. Currently, next-generation sequencing (NGS) or flow cytometry is used for tumor burden monitoring (154, 155). Yet, the complexity of the AML gene and phenotype is challenging to monitor (156). RS showed high accuracy, specificity, and sensitivity in detection, which can be used as a valuable tool for follow-up (92). Raman image combined with HCA is able to automatically discriminate and localize cellular components, such as hemoglobin. In addition, the accuracy of PCA and LDA classification models based on RS reached 98%. Furthermore, typical vibration characteristics of myeloblasts, promyelocytes (normal/abnormal), and erythroblasts were demonstrated. However, the International Working Group on Morphology of Myelodysplastic Syndrome (IWGM-MDS) reported 72–85% accuracy of manual examination (93).

In addition, a better understanding of biochemical activities and cellular response to drugs can improve treatment. In 2017, Denbigh et al. performed vibrational spectroscopy (FTIR and RS) on regular AML cells and cells treated with a combination of bezafibrate and medroxyprogesterone acetate (BaP). Spectral differences revealed a significant change in cellular lipid composition, indicating that lipid biochemistry is a significant target for BaP. The accuracy of classification was 95.23% for BaP-treated cells and 89.11% for control cells (94). In order to predict patients’ prognosis, SERS was used to measure biomolecular differences in bone marrow supernatant fluid. The findings showed differences in amino acids, saccharides, and lipids between patients with good and poor prognosis. An AML prognostic model was established after multivariate analysis of SERS results and achieved a prediction accuracy of 84.78%. (95).

Traditional diagnosis of ALL and AML mostly rely on morphology, flow cytometry, polymerase chain reaction, and gene sequencing (157, 158), but these methods are either time-consuming or costly. In contrast, RS is rapid and economical in ALL/AML diagnosis. RS also reveals biomolecular information, which provides new perspectives in ALL/AML treatment. Since ALL and AML subtypes are numerous and hard to discriminate, abundant RS information on the different subtypes of ALL/AML is needed to make a more precise diagnosis. Establishing an RS database of ALL/AML is an effective way to improve the clinical applications of RS.

### 3.4 Lymphoma

Lymphomas are one of the most common types of cancer in adolescents and can be classified into Non-Hodgkin (NHL) (90%) and Hodgkin (HL) (10%). Nearly 90% of lymphomas are derived from B-cell and T-cell, and natural killer cells (NK) origin accounts for only 10% (159). Lymphoma accounts for 22% of all cancers in patients aged between 15 and 24 years (16% of HL and 6% of NHL) (160, 161). Due to advances in treatment techniques, the overall survival five-year of lymphoma has improved from 80.4 to 93.4% for HL and 55.6 to 76.2% for NHL (162). More rapid and accurate diagnosis and classification of lymphoma are vital. The applications of RS in lymphoma are summarized in **Figure 4**.

In 2022, Katsara et al. suggested a rapid RS method for characterization and differentiation. This method provides a non-destructive strategy for early and accurate lymphoma classification (163). SERS can be a non-destructive diagnosis and staging diffuse large B-cell lymphoma (DLBCL) strategy on serum. Spectra of DLBCL in different stages were compared and provided different Raman spectral intensities. DLBCL serum samples had relatively higher intensities at Raman bands 725, 1093, 1329, 1371, and 1444 cm^−1^ (hypoxanthine, adenine, thymine, collagen, and phospholipids) and lower intensities at bands 493, 636, 888, 1003, 1133, 1580, and 1652 cm^−1^ (ergothioneine, uric acid, Tyr, lactose, Phe, acetoacetate, amide I, and α-Helix). Multivariate analysis methods were then used to establish DLBCL diagnosis and staging models. The accuracy, sensitivity, and specificity of k-nearest neighbors (kNN) classifier model was 87.3%, 92.1%, and 80.9%, respectively. As for the staging model, the discrimination accuracy of early (Stage I & II) and late (Stage III & IV) stages was 90.6% (96). In addition, the distinction between B-cell lymphoma (BCL), T-cell lymphoma (TCL), and lymph node metastasis of melanoma (Met) is vital in treatment. However, diagnosis through pathology or extensive immunohistochemistry staining is laborious (164). RS is a rapid and novel method of diagnosis. Two spectral differences were found in bands of 5-methylcytosine and adenine. The overall discriminatory accuracy between B-cell lymphoma (BCL), T-cell lymphoma (TCL), and lymph node metastasis of melanoma (Met) was 94.7% (97). In early 2003, SERS active substrates were used to detect cancer genes, such as BCL2. The SERS gene probes in this study can be used to detect DNA targets which possess the sensitivity and specificity to detect cancer genes (165).

Additionally, Agsalda-Garcia et al. performed standard RS to analyze 11 pediatric NHL and non-malignant tissue specimens from pediatric patients. However, pediatric NHL specimens consisted of 100% tumor tissues. The sensitivity of RS in samples comprised of more than just tumor remains unknown (166). Intraocular lymphoma is a special type of lymphoma that is difficult to diagnose (167). RS was selected to improve the diagnosis of intraocular lymphoma by analyzing spectra from intraocular inflammatory leukocytes and other samples using multivariate analysis. The sensitivity and specificity to discriminate between lymphoma cells and normal B cells ranged from 74–94% and 90–100% (98).

A study from 2020 reported *in vitro* blood plasma analysis of hematopoietic tumors based on RS. Spectral characteristics of plasma of DLBCL and chronic lymphocytic leukemia (CLL) were discovered, and models of DLBCL and CLL were built using orthogonal partial least squares discriminant analysis (OPLS-DA). Raman shifts at 1445 cm^-1^ and 1655 cm^-1^ were used to distinguish DLBCL and CLL, demonstrating high diagnostic sensitivity (92.86% and 80%) and specificity (100% and 92.31%) (99).

The current diagnosis of lymphoma is inclined to needle biopsy or surgical excision (168). These methods are destructive and complex. Compared to traditional diagnostic methods, RS is much simpler, and the samples for analysis are diverse and easy to acquire. Similar to other pediatric cancers, an accurate diagnosis requires the support of a large database. Studies that enlarge the RS database are needed in future to enhance the diagnosis. In addition, RS equipment is too expensive in some areas with high incidences of Burkitt lymphoma (169). Therefore, there is a need to invent economical and affordable RS equipment.

### 3.5 Glioma

Glioma is a single-celled disease that occurs anywhere in the CNS, especially in the brain and glial tissue (170, 171). It is the most common primary CNS tumor, accounting for around 30% of CNS tumors and 80% of malignant tumors (172). The World Health Organization classifies gliomas into four grades based on malignant behaviors (173). Gliomas are generally classified into two typical types: Low-grade glioma (LGG), which includes grades I and II; and high-grade glioma (HGG), which includes grades III and IV (174). LGG and HGG accounts for nearly 33% and 62% of all gliomas, respectively (175). Besides, gliomas can also be divided into various kinds of tumors, from low-risk ependyma to the most dangerous glioblastoma (GBM), based on the histological features (176). The applications of RS in glioma are summarized in **Figure 4**.

LGG is the most common glioma during childhood and accounts for over 30% of CNS tumors (177, 178). The survival rate of glioma is high (20-years overall survival of 87 ± 0.8%) since it can be surgically removed (179). However, HGG is rare but very aggressive and fatal due to its high recurrence. The incidence of HGG in children is higher than in adults (180) and has a low survival rate of about 20% (181). Thus, the clinical application of optical technologies like RS is promising. The applications of RS in glioma are summarized in **Figure 4**.

Iturrioz-Rodríguez et al. compared GBM cells with healthy human astrocytes *in vitro* using RS. Their results revealed that spectral regions ranging from 1000–1300 cm^-1^ provide sufficient information for discrimination. Raman peaks related to DNA/RNA and cytochrome C are increased in cancer cells. Their model distinguished cancer cells from healthy cells with an average accuracy of 92.5% (100). Moreover, Ma et al. obtained Raman spectra of pediatric blood plasma and used feature engineering-based classification models for prediction. After fivefold cross-validation that measures the predictive performance between models, the results showed the sensitivity, specificity, and accuracy was 98.10%, 98.19%, and 97.87% (101). Furthermore, Kowalska’s team used SERS to distinguish glioma. Their results revealed the spectral regions of Try (1450, 1278 cm^-1^), protein (1300 cm^-1^), Phe, and Amide-I (1005, 1654 cm^-1^) have the greatest influence on the study, and the accuracy of discrimination was 96% (103).

Generally, visually distinguishing cancer from normal tissue is nearly impossible. Yet, it is crucial to CNS tumor surgery since the invasive cancer cells often remain after surgery, leading to disease recurrence (182). To solve this problem, Jermyn et al. developed a handheld RS probe technique for detecting brain tumor cells. This study was conducted during surgery *in vivo* and demonstrated an accurate discrimination of normal brain from dense cancer and normal brain invaded by cancer with a specificity and sensitivity of 91% and 93% (104). Additionally, Leslie’s team evaluated the diagnostic ability of RS based on pediatric samples. They performed routine pathology tests and RS to distinguish untreated pediatric medulloblastoma, glioma, and normal brain samples. The accuracy of identification based on tissue level was 100% (105).

Due to its aggressiveness, GBM can cause death shortly after diagnosis. Aguiar et al. built a model using PCA and Euclidean distance scores to discriminate cancer tissue from normal tissue. Characteristic Raman peaks were lipid/phospholipid cholesterols and proteins. The sensitivity and specificity of *in vitro* diagnosis was 97.4% and 100% (106). Furthermore, mutations in the isocitrate dehydrogenase 1 (IDH1) gene are genetic causes of glioma, leading to metabolic changes (183). Uckermann et al. showed increased intensities in spectral bands related to DNA in IDH1 mutant glioma, whereas bands related to lipids were decreased. Results demonstrated that RS could be used in a simple, rapid, and safe IDH1 gene mutation-detecting program (184). In addition, Ricci’s team used Raman micro-spectroscopy to detect the stress response of GBM cells that adhered to a silicon substrate and showed reductions in Raman signals of cytochrome C, lipid, nucleic acid, and protein. The results demonstrated the potential of RS in studying cell processes, which can improve the treatment (107).

Similar to osteosarcoma, the diagnosis of glioma mainly depends on imageology examination. RS detection of glioma reduces the level of complexity. Moreover, RS can be used for guidance during surgery by characterizing tissue margins, leading to precise resection (185). It needs no special staining or any preparation, making real-time diagnosis possible. However, RS surely has a limited field of view. In future, this limitation can be fixed by combining RS with complementary imaging techniques (104).

## 4 Discussion and future perspectives

Pediatric cancers have significant differences compared to adult cancers. Most adult cancers are epithelial, composed of many somatic mutations, and are often influenced by environmental factors, such as smoking. In contrast, pediatric cancers are generally natural and possess few somatic mutations. Therefore, prevention and diagnosis are not as effective as in adult cancers (11, 186). Early and accurate diagnosis is crucial in pediatric cancers. Delayed diagnosis often leads to advanced diseases, complications, and increased risk of death (187–189). In some areas, the accuracy of diagnosis is limited by the lack of equipment (190–192).

RS techniques are often compared to traditional techniques. It is not meant to replace classic techniques but to fill the niches. Imagological examinations and biopsies are indispensable in pediatric cancer (193). They assist clinicians in making an accurate diagnosis. Early diagnosis of pediatric cancer is crucial since it influences the patient’s treatment and outcome. Although histological biopsy is generally applicable and is considered the gold diagnosis standard in many cancers (194), it is commonly applied after observing tumor-like structures using imagological examinations. Thus, histological biopsy cannot be applied for early cancer screening. In addition, it requires complex operations, which are highly destructive (195). In contrast, RS techniques are suitable for early diagnosis. RS is able to discriminate cancer cells from normal cells after profiling the characteristic Raman peaks. Whole blood, plasma, bone marrow supernatant fluid, and tissue samples can be used for diagnosis. Compared to laboratory tests in leukemia, RS is time-saving and accurate. During osteosarcoma or glioma surgery, precise information of margin offered by biopsies is time-consuming, and imagological examinations are unable to distinguish cancer cells from normal cells. Handheld RS techniques offer *in vivo* margin information to help tumor resection. Histological accuracy is not completely 100% as it depends on the professional level of clinicians and medical equipment. A study demonstrated the 75% accuracy of diagnosis of Burkitt lymphoma in Uganda. Most pathologists have not received specific training in the differential characteristics of NHL and HL due to limited resources (190). RS-based diagnostic models can assist pathologists in achieving higher accuracies.

Most studies are aimed at identifying cancer cells through characteristic Raman bands. They were performed *in vitro*, using samples and cells acquired from companies or patients. *In vivo* experiments are rare in pediatric cancer diagnosis. They mainly focused on evaluating therapy or guiding surgery. However, *in vivo* application of RS is vital in pediatric cancers and is the future direction of RS studies. In addition to its application in diagnosis, RS can reveal the deep interaction of drugs or biomolecules. Despite the significant differences between pediatric and adult cancers, some studies used samples from a wide age group of patients. This covers most age groups, resulting in low accuracy or sensitivity of diagnosis and misunderstanding of Raman fingerprints. There is a need to limit the age range of patients. In addition, during clinical practice, samples from patients always come with normal tissues. Thus, the accuracy, specificity, and sensitivity of models in studies that focus on analyzing 100% pure cancer cells are limited in clinical applications. The sensitivity and accuracy of RS in discriminating and classifying tissues composed of mixed cells need to be assessed.

Deep learning based on a large amount of Raman spectra is also popular nowadays. It provides a fast and accurate diagnosis of cancers. Furthermore, it can predict the aggressiveness of cancer and make better decisions for patients. However, it is essential to preprocess the spectra data before inputting it into deep learning training. This is important for selecting useful signals for deep learning and enhancing spectral features. A study aimed to classify melanocytes and melanoma by combining SERS with deep learning. Convolutional neural networks were constructed for classification and demonstrated an accuracy of over 98%. This study highlights the great potential of combining RS and deep learning in clinical applications (196). Studies have demonstrated that preprocessing of raw data can greatly affect the outcome of diagnosis (49, 197). Additionally, researchers have combined RS with other spectroscopies like IRS and FS to overcome the disadvantages of RS. They can complement the shortcomings of each other. IRS and RS were combined for oral cancer and breast cancer diagnosis (198, 199). Furthermore, RS was used in combination with Fourier Transform Infrared (FTIR) spectroscopy to identify endometrial cancer and atypical hyperplasia (200). This combination was also used in determining chemical changes in GBM (201). Besides IRS, the combination of RS with FS has been applied for breast cancer diagnosis (202).

## 5 Conclusion

Raman spectroscopy can provide molecular information on pediatric cancers, which demonstrates its potential in clinical applications of pediatric cancers. Raman fingerprints of different pediatric cancer were established for diagnosis, and diagnostic models were built and evaluated based on these characteristic Raman bands. A decent accuracy, specificity, and sensitivity indicate the potential role of RS in clinical diagnosis. As for treatment, RS can detect *in vivo* cancer cells in tumor resection margins and reveal interactions between drugs and cancer cells. In summary, Raman has the ability to provide a rapid and accurate early diagnosis of pediatric cancers, predict cancer prognosis, and improve treatment.

In order to obtain higher resolution, faster results, and better accuracy, it is necessary to develop enhanced Raman spectral databases, suitable algorithms, and advanced instruments. More studies on the applications of RS in pediatric cancer are needed to make RS a stable, effective tool against pediatric cancers.

## Author contributions

CL made crucial contributions to conception and manuscript production. CF contributed to article collection. RX contributed to figure production. BJ participated in table production. LL and YH participated in the revising of the manuscript. CT provide oversight and leadership responsibility for the research activity planning and execution. ZL provide management and coordination responsibility for the research activity planning and execution. All authors read, revised, and approved this manuscript and agreed to be responsible for allaspects of the research to ensure the data accuracy and integrity of this work.

## Glossary

**Table:**

CNS	central nervous system
RS	Raman spectroscopy
SERS	surfaced enhanced Raman spectroscopy
IRS	infrared spectroscopy
FS	fluorescence spectroscopy
PCA	principal component analysis
HCA	hierarchical cluster analysis
LDA	linear discriminant analysis
PLS	partial least squares
OS	osteosarcoma
SEER	Surveillance, Epidemiology, and End Results
VIM	vimentin
EpCAM	epithelial cell adhesion molecule
AUC	area under curve
hFoB	human fetal osteoblast
hMSC	human bone marrow-derived mesenchymal stem cells
HA	hydroxyapatite
MSC	mesenchymal stomal cells
MMP2	matrix metalloproteinase 2
MMP9	matrix metalloproteinase 9
ECRT	Extra Corporeal irradiation and Reimplantation Therapy
ALL	acute lymphoblastic leukemia
AML	acute myeloid leukemia
Tyr	tyrosine
Trp	tryptophan
Phe	phenylalanine
BaP	bezafibrate and medroxyprogesterone acetate
CML	chronic myeloid leukemia
HDL	High-density lipoprotein
LDL	low-density lipoprotein
ADA	adenosine deaminase
NHL	Non-Hodgkin lymphoma
HL	Hodgkin lymphoma
NK	natural killer
MTX	Methotrexate
IWGM-MDS	International Working Group on Morphology of Myelodysplastic Syndrome
DLBCL	diffuse large B-cell lymphoma
Met	melanoma
OPLS-DA	orthogonal partial least squares discriminant analysis
SVM	support vector machine
kNN	k-nearest neighbors
BCL	B-cell lymphoma
TCL	T-cell lymphoma
Met	melanoma
PLS-DA	k-nearest neighbors
CLL	chronic lymphocytic leukemia
AsLS	Asymmetric Least Squares analysis
LGG	low-grade glioma
HGG	high-grade glioma
GBM	glioblastoma
IDH1	isocitrate dehydrogenase 1;
